# Oxidative stress-induced diseases and tea polyphenols

**DOI:** 10.18632/oncotarget.20887

**Published:** 2017-09-14

**Authors:** Xiangbing Mao, Changsong Gu, Daiwen Chen, Bing Yu, Jun He

**Affiliations:** ^1^ Animal Nutrition Institute, Sichuan Agricultural University, Chengdu, 611130, People's Republic of China; ^2^ Key Laboratory of Animal Disease-Resistance Nutrition, Chinese Ministry of Education, Chengdu, 611130, People's Republic of China

**Keywords:** oxidative stress, tea polyphenols, antioxidant capacity, pro-oxidant capacity

## Abstract

Reactive oxide species are the middle products of normal metabolism, and play a crucial role in cell signaling transduction. On the contrary, accumulation of excess reactive oxide species results in oxidative stress that often brings multifarious impairment to cells, including decrease of ATP level in cells, elevation of cytosolic Ca^2+^, DNA damage, dysfunction of biological function in lipid bilayer and so on. These effects will finally lead to all kinds of diseases. Tea polyphenols are widely considered as a kind of excellent antioxidant agents. It can be antioxidants by directly scavenging reactive oxide species or chelating transition metals, and indirectly upregulating the activity of antioxidant enzymes. In addition, tea polyphenols have also been observed a potent pro-oxidant capacity, which directly leads to the generation of reactive oxide species, and indirectly induces apoptosis and death of cancer cells. The underlying characters of its pro-oxidant activity in some diseases is not well understood. The present review we will discuss the dual character of tea polyphenols, both antioxidant and pro-oxidant properties, in some human diseases induced by oxidative stress.

## INTRODUCTION

**Figure 1:**
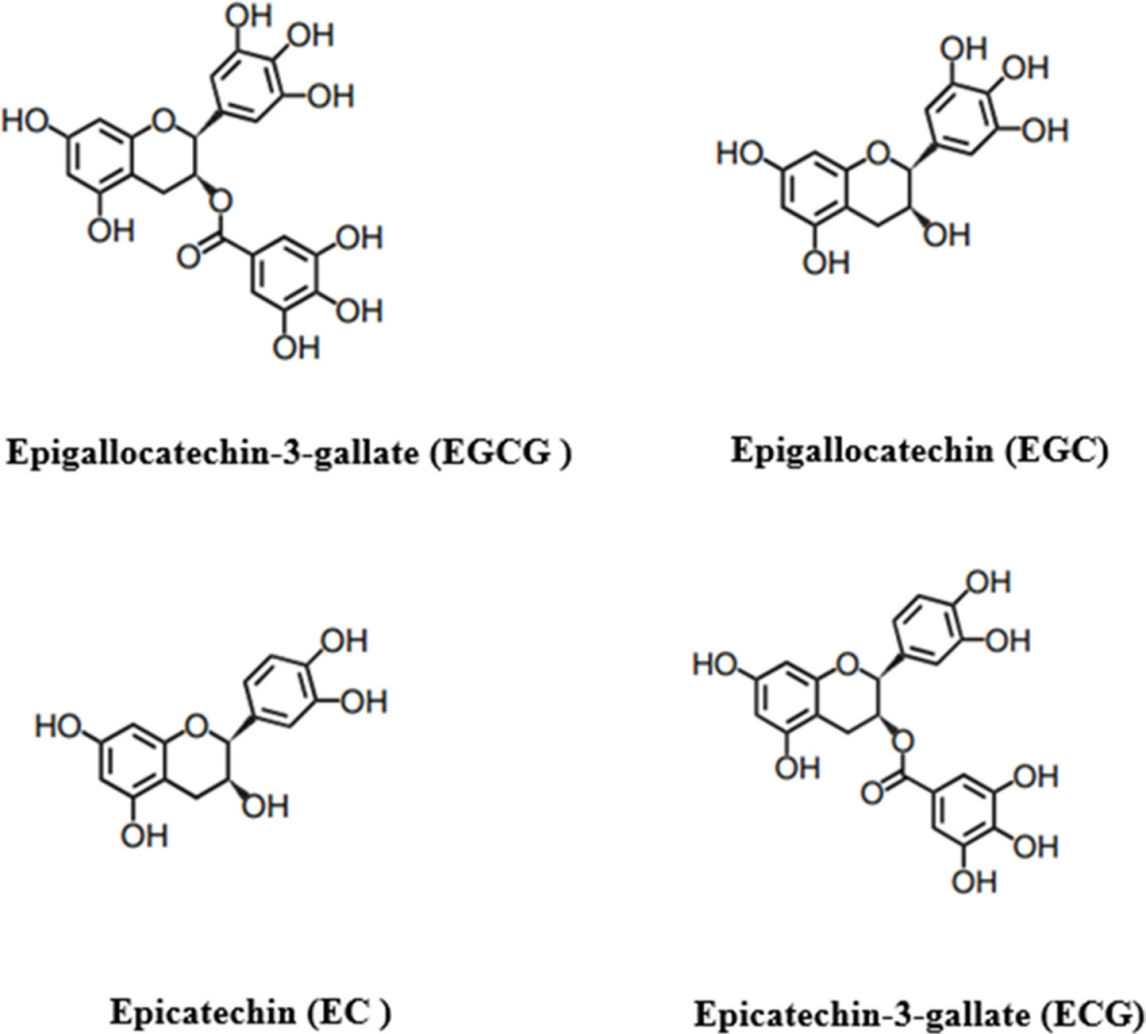
The main phenolic compounds of tea polyphenols

Tea is a popular consumed beverage worldwide [1]. According to the technology of manufacturing, tea can be classified into three major types: green tea, black tea, Oolong tea [2]. The different producing methods will lead to different chemical composition of dry leaves. Tea polyphenols, also known as catechins, is the floorboard of about 30 kinds of phenolic compounds, which are mainly including epigallocatechin-3-gallate (EGCG), epigallocatechin (EGC), epicatechin-3-gallate (ECG) and epicatechin (EC) [2] (Figure 1). And EGCG is the most abundant catechin, and may account for 50–70% of the catechins. The effective antioxidant capacity of tea polyphenols has been widely proved in lots of *in vitro* and *in vivo* studies [1, 3, 4]. This could be the critical reason that tea polyphenols, especially EGCG, have some important roles, such as anti-carcinogenic, anti-obesity, anti-inflammatory, anti-aging, anti-cancer, anti-virulence, anti-diabetic, anti-bacterial and neuro-protective effects [5–12].

The recent study have indicated that many clinical diseases will occur with oxidative damage. As an excellent antioxidant, tea polyphenols can be used to deal with these diseases via resisting oxidative damage in the body [5–12]. However, some experiments showed that tea polyphenols also act as inhibitor in cancer cells through inducing the generation of reactive oxide species (ROS) and apoptosis, and impacting the cell signaling pathway [8, 13–19]. In the present review, we will mainly discuss the antioxidant capacity of tea polyphenols and its dual characters in regulating the redox state in cells, and pay attention to summarize the possible mechanisms of these effects. This review is aiming at stimulating more researches into the role of tea polyphenols in oxidation state, and the potential mechanism for cancer prevention and cure. An overall understanding of the chemical property and biological function of tea polyphenols will be essential for understanding their ultimate virtue in preventive chronic diseases, including cancers.

### Free radicals and oxidative stress

**Figure 2:**
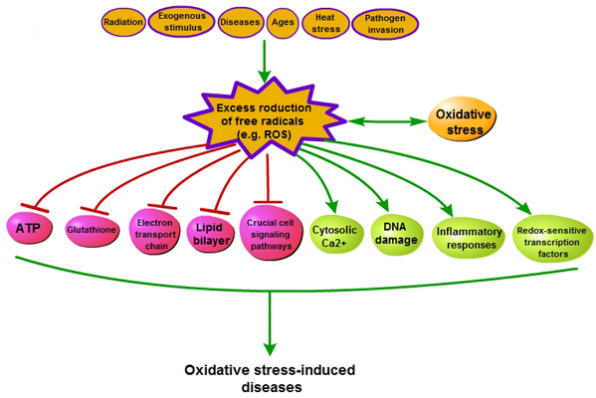
The process of oxidative stress-induced diseases

Oxidative stress results from massive accumulation of reaction oxygen species (ROS) that is induced by a wide range of factors including radiation, pathogen invasion (hypersensitive reaction), ages, disease and heat stress [20–23] (Figure 2). Generally, ROS production (Table 1) is a physiological process that take place in every aerobic organism [24]. For example, cells release superoxide and hydrogen peroxide from mitochondria in the process of ATP formation [25]. However, development and effects of oxidative stress mainly depend on the capabilities of the organism, alone or with intervention from outside, to keep the dynamic balance of redox state. Thus, oxidative stress has been defined as a disturbance in the dynamically balance between the ROS generation and the antioxidant capacity [26], which leads to the production of an array of free radicals [24]. This often brings multifarious impairment to cells, including decrease of ATP level in cells, elevation of cytosolic Ca2^+^, DNA damage, dysfunction of biological function in lipid bilayer, decrease of the glutathione level, impairment of electron transport chain, regulation of gene expression through activation of redox-sensitive transcription factors, modulation of inflammatory responses, and change of crucial cell signaling pathways [5, 27–32]. These effects could lead to all kinds of diseases [21, 28–34].

**Table 1:** Major reaction of oxygen species

Oxygen free radicals	RNS	Non-radical oxidant	Iron-oxygen complexes	Organic free radicals
O_2_	NO^·^	H_2_O_2_	Fe = O^2+^	R-O
O_2_^·−^	NO_2_^·^	ONOOH	Fe = O^3+^	R-OO
^·^OH		HCLO		^·^QH
HO_2_^·^		HOSCN		

### Oxidative stress and human disease

Many studies indicate that ROS are involved in a good deal of physiological and pathphysiological process [5–12, 35]. The appropriate level of ROS plays a significant role as regulatory mediators in the cell signaling processes of differentiation, proliferation, apoptosis, immunity, defense against microorganism, melanogenesis and aging [36–40]. Conversely, the high level of ROS is dangerous for living organism as they are detrimental to the major cellular components. When the living organisms stay at oxidative stress state for a long time, the disease will occur in various system (Table 2).

**Table 2:** Diseases that have been linked to oxidative stress

Disease	Reference
Neurological Disease	
Alzheimer's Disease	38, 49
Parkinson's Disease	32, 37, 83
Huntington's Disease	84
Wilson Disease	85
Cancer	33, 39, 50, 53, 61
Aging	21
Vascular Disease	13 58
Pulmonary Disease	69
Diabets	15 18 60
Skin Disease	88
Chronic kidney Disease	36, 89
Inflammation	27, 66, 68, 69, 72
Obesity	90

### Oxidative stress and neurodegenerative disease

**Figure 3:**
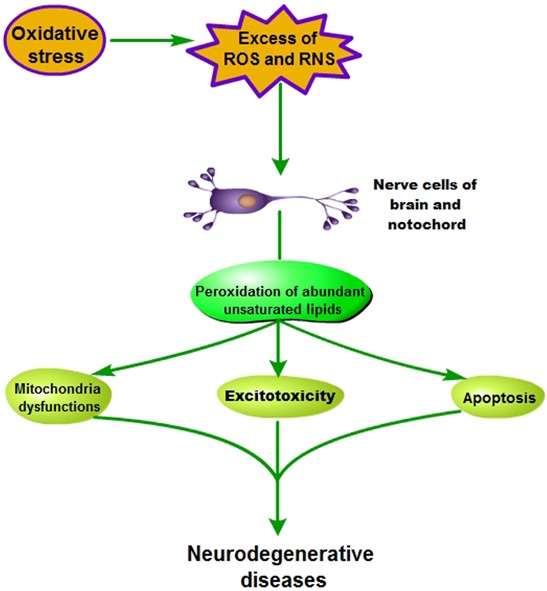
Pathogenesis of oxidative stress-induced neurodegenerative diseases

Several researches have shown that human brain is eccentrically sensitive to oxidative damage [36, 38] (Figure 3). Brain consumes an inordinate fraction (20%) of total oxygen consumption for its relatively small weight (2%), but it doesn't enrich antioxidant defenses [28]. Antioxidant activity in brain is lower than that in other tissues [41], for example, about 10% less than liver [42]. However, neuronal biochemical composition is mainly sensitive to ROS, which is due that it contains abundant unsaturated lipids that are labile to peroxidation and oxidative modification [43].

With the oxidative stress forming, human nerve cells of brain and notochord lead to either functional loss or dysfunction perception, and further mitochondria dysfunctions, excitotoxicity and apoptosis were observed during neurodegenerative diseases [37, 38, 41]. Thus, oxidative stress, inducing production of a plenty of ROS and reactive nitrogen species (RNS), is implicated as the pathogenesis of many neurodegenerative diseases, such as Parkinson's disease, Alzheimer's disease, mutiple sclerosis and amyolotrophic lateral sclerosis [44].

### Oxidative stress and carcinogenesis

**Figure 4:**
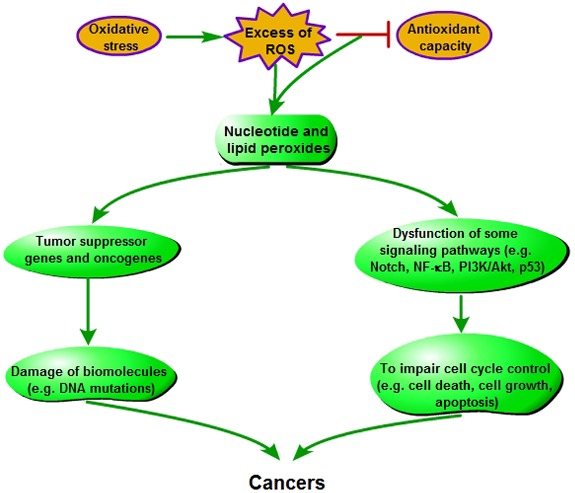
Pathogenesis of oxidative stress-induced carcinogenesis

Oxidative stress derived from excess ROS production can lead to carcinogenesis in normal cells (Figure 4). ROS inducing nucleotide and lipid peroxides will directly and indirectly aggravate damage of biomolecules, including DNA (such as mtDNA and genomic DNA) in chain reactions [28]. Besides impairing the genes that are related to cell cycle control or DNA damage pathway [45], ROS-induced DNA damage gives rise to mutations involved in tumor suppressor genes or oncogens [34]. If these damages can not be properly repaired, DNA mutations will occur, which further induces cancer in the end. This is an oncogenic mechanism of oxidative stress. Several previous clinical studies have also implicated ROS exposition with an increased risk of cancer [43, 46]. Moreover, researches on various types of cancer show a possible link between the low activity of superoxide dismutase and the high level of hydroxylated DNA base [47–50].

In addition, it has been definitively recognized that apoptosis plays an important role in controlling the tumor expansion [51]. Many studies indicated that cancers, including lymphomas, p53-mutation carcinmas and some hormone-dependent tumors (such as breast, prostate, ovarian, pancreatic and colon cancers), have the closely association with the inhibiton of apoptosis [52]. The activities of several genes known to be influential in cancer progression have been indentified as modulating cell death, especially Bcl-2, fas, and p53 [53]. Meanwhile, the inhibiton of apoptosis in tumor progression can involve some signaling pathways, such as Notch, nuclear factor-κB (NF-κB), protein kinase B (Akt) and p53 [51, 54, 55]. Researchers indicated that ROS could activate the PI3K-dependent Akt counteracted by phosphatase through inhibiting phosphatase and tensin homolog deleted from chromosome 10 (PTEN), and then Akt may foster tumorigenesis [54, 55]. Akt is an inhibitor of apoptosis via triggering the NF-κB activity and regulating pro-apoptotic molecules (such as caspase-9 and Bcl-2). Moreover, Akt affects the nuclear translocation of ubiquitin ligase MDM2, which inhibits p53-mediated apoptosis [56]. It also has been reported that there is cross-talk between Notch-1 and another major cell growth and apoptotic regulatory pathway, and the downregulation of Notch-1 resulted in the increasing cell growth inhibition and apoptosis [51].

### Oxidative stress and inflammation

**Figure 5:**
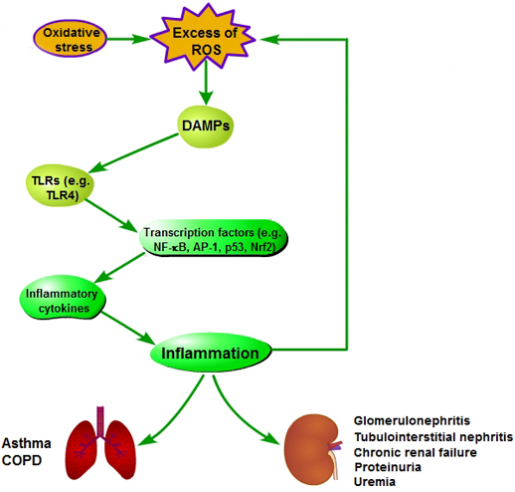
Pathogenesis of oxidative stress-induced inflammation

The persistent oxidative stress can lead to chornic inflammation [57] (Figure 5). The relative mechanisms involve the activation of many transcription factors, such as NF-κB, activator protein-1 (AP-1), p53 and Nrf2, which will lead to the expression of more than 500 genes (including inflammatory cytokines), and finally trigger inflammation [46]. During oxidative stress, ROS may promote the release of damage-associated molecular pattern molecules (DAMPs) in the damaged or apoptotic cells, which stimulates toll-like receptors (TLRs) signaling, such as TLR4, in immune cells. This will induce the production of inflammatory factors, and trigger TLR4-mediated inflammation [54]. However, during inflammation, mast cells and leukocytes are recruited to the damage site. This will lead to a ‘respiratory burst’ derived from increasing oxygen uptake, and then ROS is further released and accumulated at the damage site [55, 56]. Thus, there is a vicious circle between oxidative stress and inflammation. These also demonstrate that oxidative stress and inflammation are closely related pathophysiological processes, and both are stimultaneously found in many pathological conditions [39, 57, 58].

The inflammation derived from oxidative stress in different tissues and organisms will induce different diseases. Inflammatory lung diseases, such as asthma and chronic obstructive pulmonary disease (COPD), are characterized by systemic and local chronic inflammation and oxidative stress [59]. Oxidative stress and inflammation also play an important role in lots of renal diseases, such as glomerulonephritis, tubulointerstitial nephritis, chronic renal failure, proteinuria and uremia [60, 61].

### Oxidative stress and some other diseases

Oxidative stress relates to the generation and development of some other diseases. Obesity may induce systemic oxidative stress, and oxidative stress is associated with an irregular production of adipokines that will further induce the development of metabolic syndrome [62]. Oxidative stress also plays a key role in the pathogensis of diabetes and its complications, and then insulin resistance and beta-cell dysfunction that are two central events in the pathophysiology of type 2 diabetes have been linked to redox imbalance [18, 63, 64]. In addition, the level of oxidative stress is relative with aging [65]. Oxidative stress has been found to implicate with age-related macular degeneration and cataracts by altering various cell types in the human eye [66], and excess ROS production can lead to cross-link and aggreation of the crystalline proteins in the lens, which will cause the formation of cataracts [67]. Besides these, nephrotoxicity resulted from some drugs is mainly due to oxidative stress via lipid peroxidation in kidney [68].

### Tea polyphenols and diseases triggered by oxidative stress

Green tea consumed within a balance diet is able to improve the organism redox status, rescue cells from oxidative damage, and limit the risk of various degenerative diseases associated to oxidative stress [15, 69–71]. The *in vitro* and *in vivo* studies indicate that polyphenols are derived from tea may have the bioactivity to affect the pathogenesis of several chornic disease [4, 12, 71]. Tea polyphenols curing diseases has been partially attributed to the antioxidative capacity. And, in all tea polyphenols, EGCG is mainly responsible for antioxidant capacity.

Many studies show that administration of tea polyphenols can limit carcinogenesis, neurodegenerative diseases, inflammation, aging and renal disease [15, 19, 66, 67, 72]. Most of these diseases are associated with the damage of DNA, proteins and lipids caused by oxidative stress. Tea polyphenols can help to limit this damage via two ways. One is that it can directly act on ROS, and the other is that it may stimulate endogenous defence system [73].

### The mechanism of tea polyphenols curing diseases triggered by oxidative stress

**Figure 6:**
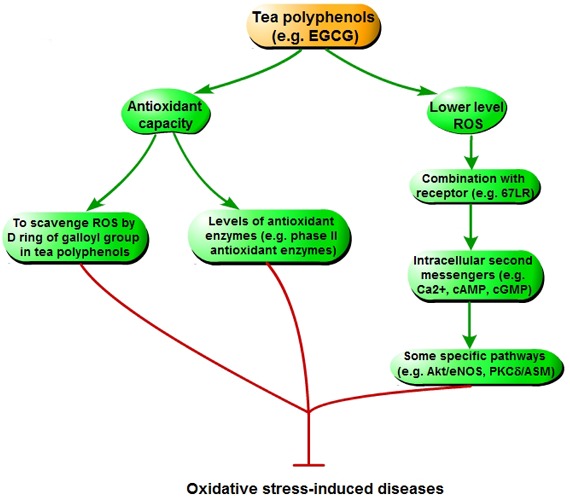
The mechanism of tea polyphenols curing diseases triggered by oxidative stress

Tea polyphenols can relieve the oxidative stress by scavenging ROS and generating more stable phenolic radicals *in vivo* and *in vitro* (Figure 6). The ability of EGCG scavenging radicals is mainly derived from the D ring in the galloyl group of its structure [17]. Following analysis of electron paramagnetic resonance spectroscopy, the .OH and O^2–^ are scavenged by EGCG mainly through oxidating the D ring of galloyl group [22, 73]. In addition, tea polyphenols also have indirect antioxidant effects. Many studies have shown that treatment of tea polyphenols may increase the levels of phase II antioxidant enzymes, such as glutathione peroxidase and reductase, glutathione S-transferase, catalase, quinone reductase, and superoxide dismutase in different organs (including liver, small intestine, lung, skin, brain, prostate and oral cavity) of rodents [74–78]. Administration of tea polyphenols can decrease the level of 8-oxoguanine that is a reliable marker for DNA damage induced by oxidative stress, and inhibit DNA oxidative damage via reducing the expression of cytochormes P450 [79–82]. Therefore, tea polyphenols may cure oxidative stress-relative diseases via increasing antioxidant capacity that can relieve the oxidative damage [25, 26, 83–85].

Moreover, there are some other indirect ways of tea polyphenols inhibiting disease induced by oxidative stress. In cell cuture experiment, EGCG is mainly found in the cytosol [86], which indicates that biological function of EGCG may occur through EGCG metabolites or interaction between EGCG and intracelluar molecules. EGCG producing the lower level of ROS, including hydrogen peroxide, will act as a messenger molecule for downstream signaling pathway [87]. However, via combining with a specific receptor (67 kD laminin receptor, 67LR), EGCG also increases some other intracellular second messengers, such as Ca^2+^, cAMP, and cGMP [88–90]. These messengers, espeicially cGMP, can activate some specific pathways, such as protein kinase B/endothelial nitric oxide synthase (Akt/eNOS) and protein kinase Cδ/acidic sphingomyelinase (PKCδ/ASM). This inhibits oxidative stress, and cures the relative diseases (including cardiovascular disease, cancer and neurodegenerative disease) [25].

### Some special pathways involved in tea polyphenols inhibiting cancers

**Figure 7:**
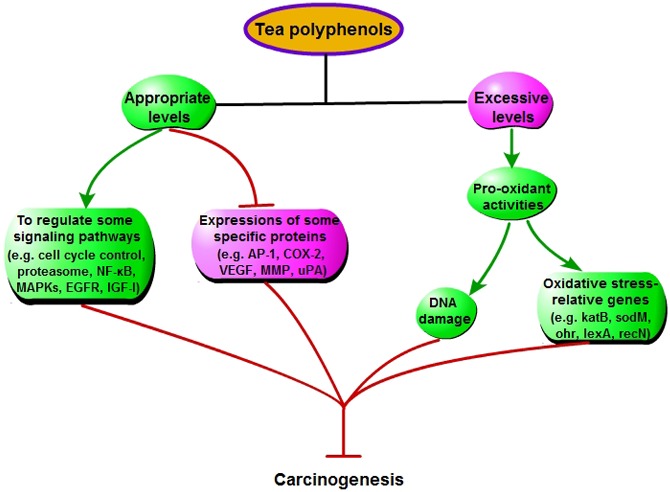
Some special mechanisms that tea polyphenols inhibit cancers, except increasing antioxidant capacity

Tea and tea extractive regulating some pathways can also inhibit tumorigenesis in oral, esophageal, forestomach, stomach, intestinal, colon, skin, liver, bladder, prostate and breast cancers [3, 91–93] (Figure 7). (1) Tea polyphenols can induce apoptosis and cell cycle arrest of cancer cells via regulating caspase-3 activation, nuclear condensation, and the expression of Bax, Bcl-2, p21 and p27 (p27Kip1). (2) Tea polyphenols, especially EGCG, can modulate NF-κB, MAPKs, proteasome, epidermal growth factor receptor (EGFR)-mediated, and insulin-like growth factor-I-mediated signaling pathway in cancers. (3) The expressions of some proteins, including AP-1, cyclooxygenase-2 (COX-2), vascular endothelial growth factor (VEGF), matrix metalloproteinase (MMP) and urokinase-plasminogen activator (uPA), are inhibited by tea polyphenols in cancers.

### Pro-oxidant activities of tea polyphenols

Although tea polyphenols have generally been regarded as antioxidant, some evidence for its pro-oxidant property is interesting. Tea polyphenols are unstable, and undergo autooxidative reactions resulting in ROS production *in vivo* and *in vitro* (like H_2_O_2_) [94, 95]. Some reports have shown that tea polyphenols are the apoptosis-inducing agent presenting in the popular beverage [96], increase phosphorylation of histone 2A.X (γH2A.X) that is a marker of oxidative DNA damage, and enhance the expression of oxidative stress-relative genes, such as *kat*B, *sod*M, *ohr*, *lex*A and *rec*N. This can be an important mechanism that tea polyphenols inducing the death of cells via the pro-oxidant property may inhibit carcinogenesis [3] (Figure 7). In the *in vitro* experiments, tea polyphenols, such as EGCG and EGC, inhibiting prostate cancer cells is a dose-dependent and pH-value-dependent process [97, 98]. Besides these, the recent study have also shown that, compared with normal cells, cancer cells are more sensitive to pro-oxidant activity of tea polyphenols [99].

All the time, tea polyphenols are generally regarded as health promotion agents. However, the high dose of tea polyphenols triggering pro-oxidant activities exists the potential toxic effects in normal hepatocyte and livers, and this cytotoxicity is time- and dose-dependent [100, 101]. Thus, there is a hepatoxicity risk of tea polyphenols in humans.

### The different effect of tea polyphenols derived from different teas on antioxidant status

Recent studies have shown that tea polyphenols derived from different teas have different effect on oxidative stress-induced diseases [102–104]. Nowadays, there are three main types of tea consumed by human, including green tea, Oolong tea and black tea. Oolong tea is manufactured by a partial oxidation of the leaf, which is intermediate between the processes for green and black tea [105]. Green and black teas have strong antioxidant property in *in vivo* and *in vitro* studies, and green tea has higher antioxidant capacity than fermented teas, such as Oolong tea and black tea [106, 107]. Based on Ferric reducing antioxidant power assay, the lower antioxidant capacity in fermented teas can be due to the decreased content of polyphenols in the fermentation process [108]. However, by using oxygen-radical absorbance capacity assay and Trolox equivalent antioxidant capacity assay in the *in vitro* studies, black tea extract has comparable antioxidant capacity to green tea extract [109, 110]. The further study showed that black tea extract could protect tissues against oxidative damage derived from lipid peroxidation, and are more effective than green tea extract in scavenging superoxide anion [111]. In addition, Oolong tea extract has higher antioxidant capacity and lipoxygenase inhibitory activity than black tea extract [105], but the biological effect of Oolong tea remains unclear.

The different effect of polyphenols derived from three teas on antioxidant status could be associated with the concentration of monomeric and polymers polyphenols. Black tea contains lower amounts of monomeric polyphenols and higher concentrations of polymers, compared with green tea [112, 113]. Oolong tea are partially oxidized, which leads to an intermediate tea with a lower concentration of polymeric polyphenols and higher concentrations of EGCG, compared with black tea [113].

## CONCLUSIONS

Tea is a popular, socially accepted, safe and healthy beverage that is widely consumed around the world. Tea is beneficial to prevent and cure a variety of diseases related to oxidative stress. As one of mainly active compounds in tea, tea polyphenols act as an excellent antioxidant through regulating both endogenous and exogenous antioxidant mechanism, which have been clearly demonstrated in the *in vitro* and *in vivo* studies. In addition, some studies have indicated that tea polyphenols have pro-oxidant activity, which can reduce the risk of some types of cancer via ROS-mediated cancer cell death. Although there are a plenty of important results of tea polyphenols protecting the body via regulating the redox status, some further studies, such as its specific mechanism of anti-chronic diseases, need to be done. And the *in vivo* evidences about the mechanism of its anti-cancer activity should also be further afforded.
